# Methyl Brevifolincarboxylate Attenuates Free Fatty Acid-Induced Lipid Metabolism and Inflammation in Hepatocytes through AMPK/NF-κB Signaling Pathway

**DOI:** 10.3390/ijms221810062

**Published:** 2021-09-17

**Authors:** Madamanchi Geethangili, Chiao-Wei Lin, Harry J. Mersmann, Shih-Torng Ding

**Affiliations:** Department of Animal Science and Technology, National Taiwan University, Taipei 10672, Taiwan; geetha81@ntu.edu.tw (M.G.); d04642002@ntu.edu.tw (C.-W.L.); mersmann@msn.com (H.J.M.)

**Keywords:** methyl brevifolincarboxylate, NAFLD, TG, de novo lipogenesis, lipid oxidation, inflammation

## Abstract

The prevalence of non-alcoholic fatty liver disease (NAFLD) is one of the leading causes of chronic liver diseases worldwide. This study examined the potential protective effects of a naturally occurring polyphenolic compound, methyl brevifolincarboxylate (MBC) on fatty liver injury in vitro. The results showed that MBC at its non-cytotoxic concentrations, reduced lipid droplet accumulation and triglyceride (TG) levels in the oleic acid (OA)-treated human hepatocarcinoma cell line, SK-HEP-1 and murine primary hepatocytes. In OA-treated SK-HEP-1 cells and primary murine hepatocytes, MBC attenuated the mRNA expression levels of the de novo lipogenesis molecules, acetyl-coenzyme A carboxylase (*Acc1*), fatty acid synthase (*Fasn*) and sterol regulatory element binding protein 1c (*Srebp1c*). MBC promoted the lipid oxidation factor peroxisome proliferator activated receptor-α (*Pparα*), and its target genes, carnitine palmitoyl transferase 1 (*Cpt1*) and acyl-coenzyme A oxidase 1 (*Acox1*) in both the SK-HEP-1 cells and primary murine hepatocytes. The mRNA results were further supported by the attenuated protein expression of lipogenesis and lipid oxidation molecules in OA-treated SK-HEP-1 cells. The MBC increased the expression of AMP activated protein kinase (AMPK) phosphorylation. On the other hand, MBC treatment dampened the inflammatory mediator’s, tumor necrosis factor (*TNF*)-α, interleukin-6 (*IL-6*), *IL-8*, and *IL-1β* secretion, and nuclear factor (NF)-κB expression (mRNA and protein) through reduced reactive oxygen species production in OA-treated SK-HEP-1 cells. Taken together, our results demonstrated that MBC possessed potential protective effects against NAFLD in vitro by amelioration of lipid metabolism and inflammatory markers through the AMPK/NF-κB signaling pathway.

## 1. Introduction

Nonalcoholic fatty liver disease (NAFLD) is one of the most commonly occurring chronic liver disorders [1]. It is caused by inadequate lipid metabolism without alcohol consumption [2]. In general, lipid metabolism is regulated through various pathways such as uptake of circulating lipids, de novo lipogenesis, fatty acid oxidation (lipolysis), and secretion [2]. However, dysregulated lipid metabolism results in excessive lipid accumulation in the form of triglycerides (TG) in the liver, that plays a major role in the development of NAFLD [2]. The development of NAFLD proceeds through a spectrum of disturbances, ranging from hepatic lipid accumulation (steatosis) to nonalcoholic steatohepatitis (NASH), liver fibrosis, cirrhosis, and hepatocellular carcinoma (HCC) [1]. Moreover, NAFLD is also associated with metabolic diseases such as hyperlipidemia, chronic kidney disease (CKD), insulin resistance, type 2 diabetes and cardiovascular disorders [3]. In recent years, the prevalence of NAFLD has increased substantially across continents, and become a worldwide burden on health [4]. It is estimated to affect around one billion individuals worldwide with different burdens according to sex, ethnicity, and age [4]. Until now, there is no approved drug by the US FDA to treat patients with NAFLD [4]. The therapeutic trial agents such as insulin sensitizers (metformin, glitazones, pioglitazone), nuclear receptor agonists (elafibranor, obeticholic acid, GFT505), and glucagon-like peptide-1 receptor agonists are still in the clinical trial process against NAFLD [5]. Thus, there is a need to develop novel therapeutic agents against NAFLD, and to elucidate possible molecular mechanisms.

Hepatic lipid accumulation activates and/or inhibits the molecular pathways associated with NAFLD [6]. Among these, adenosine monophosphate-activated protein kinase (AMPK) is an important kinase for lipid metabolism [7]. AMPK coordinates the intracellular lipid metabolism through the transcriptional regulation of key de novo lipogenesis as well as fatty acid oxidation factors [7]. Activated AMPK through its Thr172 phosphorylation leads to decreased expression and/or activity of lipogenic molecules, such as acetyl-CoA carboxylase 1 (ACC1), fatty acid synthase (FASN) and sterol regulatory element-binding protein (SREBP)-1c, and increased the expression of the lipid oxidation factor, peroxisome proliferator activated receptor (PPAR)-α [7]. It is known that elevated inflammation through secretion of inflammatory mediators, such as interleukin (IL)-6, IL-8, IL-1β, tumor necrosis factor (TNF)-α and activation of nuclear factor (NF)-κB promotes the occurrence and development of NAFLD [7]. Therefore, phosphorylation of AMPK (Thr172), and attenuation of inflammation are attractive targets for the prevention of NAFLD.

**Figure 1:**
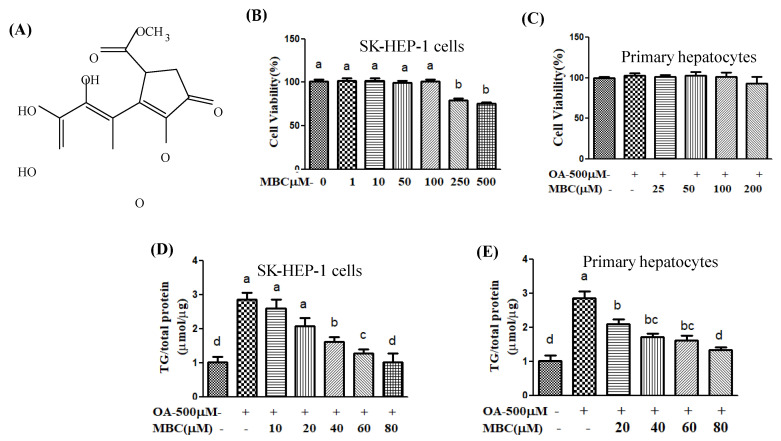
(**A**) Chemical structure of methyl brevifolincarboxylate (MBC). (**B**,**C**) Effect of MBC on viability of SK-HEP-1 cells and murine primary hepatocytes, respectively, as determined by Alamar Blue assay. The absorbance was measured at 570 and 600 nm. The cell survival rate (CSR) was calculated on basis of OD. (**D**,**E**) Effect of MBC on triglyceride (TG) levels in OA-treated SK-HEP-1 cells and primary murine hepatocytes, respectively. Total TG was analyzed using Randox Triglyceride Assay kit. Results were presented as the mean ± SD of three independent experiments. Data bars with similar let ers were not significantly different (*p* ≤ 0.05).

Natural herbal preparations and their bioactive metabolites show positive therapeutic effects against NAFLD [8]. The common plant derivatives, polyphenols are beneficial for metabolic diseases including NAFLD [8]. The hydroxyl (-OH), and/or ketone (-C=O) groups of polyphenols serve as active sites to bind with biomolecules and to exhibit therapeutic potential against NAFLD [8]. Methyl brevifolincarboxylate (MBC, Figure 1A), is a natural polyphenolic compound, isolated from various traditional herbal medicines including *Phyllanthus urinaria* [9], *P. niruri* [10], *Potentilla argentea* [11], *Geranium carolinianum* [12] and *Canarium album* [13]. The MBC exhibits a wide variety of pharmacological effects including anti-inflammatory and anticancer [9], vasorelaxant effects through inhibition of platelet aggregation [10] and anticancer activity through inhibition of topoisomerase I/II [11]. A recent study reports that MBC showed inhibitory activity against influenza virus [13]. However, the pharmacological action and mechanism of MBC on NAFLD, has not yet been reported. As part of our study program to identify novel therapeutic agents, the present study examined the in vitro anti-NAFLD effects of MBC in NAFLD-associated HCC cell line SK-HEP-1 cells and primary murine hepatocytes, treated by oleic acid (OA).

## 2. Results

### 2.1. Effect of MBC on Lipid Accumulation and TG Levels in OA-Treated SK-HEP-1 Cells and Primary Murine Hepatocytes

The effect of OA (a monounsaturated fatty acid) and MBC (Figure 1A), on the cell viability of human HCC cell line SK-HEP-1 cells and primary murine hepatocytes was determined using the Alamar Blue assay. We treated SK-HEP-1 cells with various concentrations of MBC (1, 10, 50, 100, 200, or 500 µM) or OA (0.1, 0.2, 0.5, 0.8, 1 mM) for 48 h. Similarly, primary murine hepatocytes were treated with MBC (25, 50, 100, or 200 µM) or OA (500 µM) for 48 h. The results indicated that the viability of SK-HEP-1 cells treated with 250 µM or 500 µM of MBC was reduced (Figure 1B). The tested concentrations (25, 50, 100, or 200 µM) of MBC does not noticeably affect the viability of primary hepatocytes (Figure 1C). The OA at concentration of 0.8, and 1 mM significantly reduced cells viability (data not shown). However, the compounds OA and MBC did not affect the cell viability of the SK-HEP-1 cells and primary murine hepatocytes up to their concentrations of 0.5 mM and 80 µM, respectively. Therefore, these non-cytotoxic concentrations were used in the subsequent experiments.

In order to evaluate the effect of MBC treatment on TG levels, the cellular TG contents (in cell lysates) were measured using a TG biochemical kit. The intracellular TG levels in SK-HEP-1 cells and primary murine hepatocytes were about three times higher after 0.5 mM of OA-treatment for 48 h, than in the control cells (Figure 1D,E). However, the compound MBC at concentration of 10, 20, 40, 60 and 80 µM, reduced the TG levels by 22%, 36%, 51%, 77% and 88%, respectively as compared with control cells (Figure 1D). We also observed that MBC dose-dependently (20, 40, 60, or 80 µM) alleviated the OA-induced TG levels in primary murine hepatocytes (Figure 1E).

**Figure 2:**
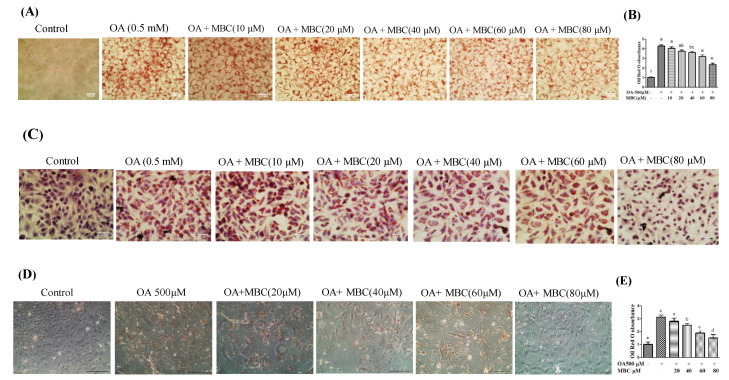
Effect of methyl brevifolincarboxylate (MBC) on OA-induced intracellular lipid accumulation in SK-HEP-1 cells and primary murine hepatocytes. Cells were plated into 12 well plate dishes and treated with different doses of MBC (as indicated in the figure) in the presence of OA (0.5 mM) for 48 h. (**A**) SK-HEP-1 cells were stained with Oil Red O. (**B**) Quantitative data for lipid accumulation in SK-HEP-1 cells (Oil Red O staining). (**C**) H&E staining of OA and MBC-treated SK-HEP-1 cells. (**D**) Primary murine hepatocytes were stained with Oil Red O. (**E**) Quantitative data for lipid accumulation in primary murine hepatocytes (Oil Red O staining). Scale bar = 100 µm. The results were expressed as mean ± SD from three independent experiments. Data bars with similar letters were not significantly different (*p* < 0.05).

The Oil Red O staining method was used to evaluate the effect of MBC on lipid accumulation in OA-treated SK-HEP-1 cells and primary murine hepatocytes. Coordinated with the results of TG levels, Oil Red O staining showed an elevated lipid droplet formation in SK-HEP-1 cells as well as primary murine hepatocytes after 0.5 mM of OA-treatment for 48 h (Figure 2A,D, respectively). The compound MBC dose-dependently (10, 20, 40, 60 and 80 µM) reduced the droplet formation in OA-treated SK-HEP-1 cells (Figure 2A). The absorbance values were reduced by 22%, 29%, 37% 46%, and 61% with the MBC treatment at 10, 20, 40, 60 and 80 µM, respectively (Figure 2B). In addition, the histomorphological analysis with hematoxylin and eosin (H&E) staining revealed that OA-induced the density of ballooned (enlarged) SK-HEP-1 cells (Figure 2C). These alterations were dose-dependently (10, 20, 40, 60 and 80 µM) ameliorated by MBC treatment (Figure 2C). Similar results were observed in primary murine hepatocytes treated with various doses of MBC (Figure 2D). Treatment with MBC for 48 h, dose-dependently (20, 40, 60, or 80 µM) reduced the accumulation of lipid droplets in OA-treated hepatocytes (Figure 2D,E). Therefore, MBC attenuated lipid deposition in the NAFLD in vitro model.

### 2.2. Effect of MBC on Lipid Metabolism Molecules Activity and Expression in OA-Treated Hepatocytes

**Figure 3:**
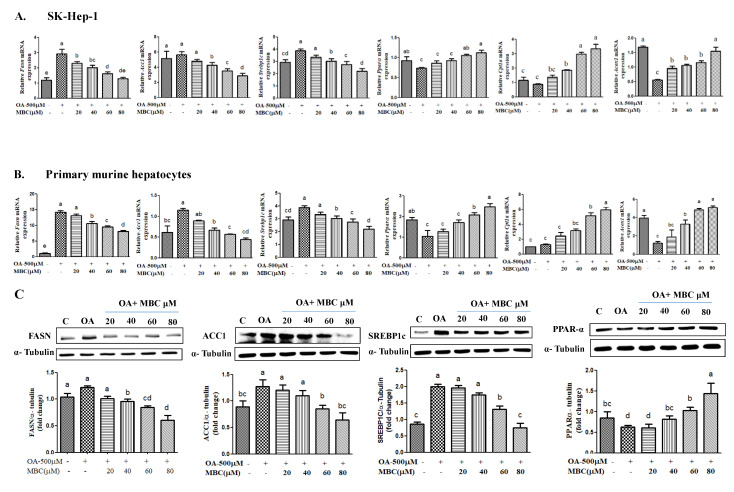

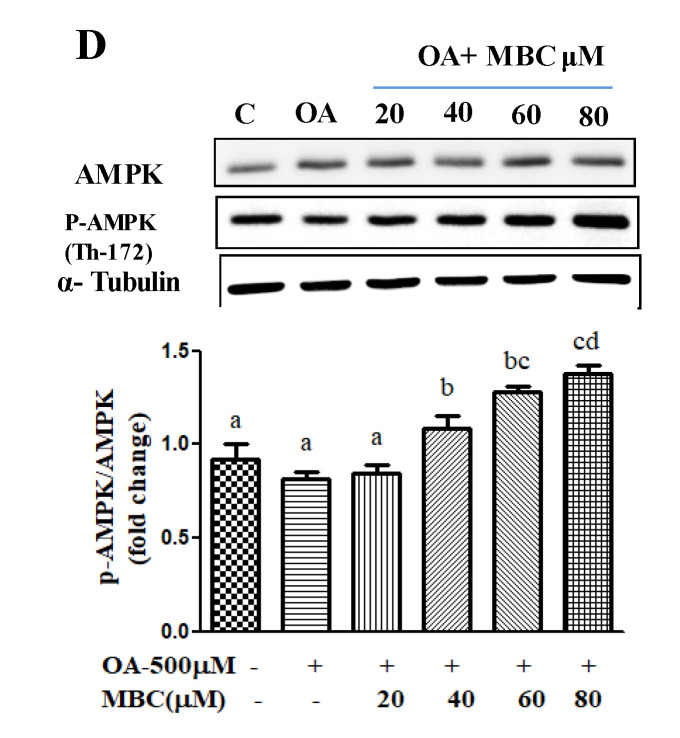
Effect of methyl brevifolincarboxylate (MBC) on expression of lipogenesis and lipid oxidation mRNA and proteins in OA-treated SK-HEP-1 cells (**A**), and primary murine hepatocytes (**B**). Cells were treated with 0.5 mM of OA, and different concentrations of MBC (0, 20, 40, 60, and 80μM) for 48 h. Total RNA was isolated using a GENEzol reagent and mRNA was measured using qRT-PCR. Target gene mRNA levels were normalized to a reference gene β-actin. (**C**) Protein expression of FASN, ACC1, SREBP-1c, PPAR-α and (**D**) p-AMPK were detected by Western blot. All results are expressed as mean ± SD of three independent experiments. Data bars with similar letters were not significantly different (*p* ≤ 0.05).

To determine the underlying possible molecular mechanisms for MBC reduced lipid accumulation and TG levels in OA-treated SK-HEP-1 cells and primary murine hepatocytes, we examined the effect of MBC on the messenger RNA (mRNA) levels of key genes associated with de novo lipogenesis using real-time PCR. The results revealed that the mRNA expression levels of the lipogenesis genes *Acc1*, *Fasn*, and *Srebp1c* were increased in SK-HEP-1 cells after 0.5 mM of OA-treatment for 48 h compared to control cells (Figure 3A). The MBC treatment for 48 h dose-dependently (20, 40, 60 and 80 µM) down regulated the expression of *Acc1*, *Fasn*, and *Srebp1c* (Figure 3A). The OA-treatment caused a down-regulation of the lipid oxidation gene *Pparα* in SK-HEP-1 cells. The MBC treatment dose-dependently (20, 40, 60 and 80 µM) increased the *Pparα* expression in OA-treated SK-HEP-1 cells (Figure 3A). Similarly, OA-treatment decreased the *Cpt1a* mRNA level in SK-HEP-1 cells, which were dose-dependently attenuated by MBC treatment (Figure 3A). Hepatic *Acox1* mRNA levels were also decreased by the OA-treatment, and were dose-dependently augmented by MBC (Figure 3A). These results were further confirmed in primary murine hepatocytes (Figure 3B). The mRNA levels of lipogenic genes *Acc1*, *Fasn*, and *Srebp1c* were noticeably increased by OA exposure in primary hepatocytes, and MBC treatment attenuated this increase in a dose-dependent (20, 40, 60 or 80 µM) manner (Figure 3B). Similarly, MBC treatments dose-dependently increased the mRNA levels of lipid oxidation gene *Pparα* and its target genes *Cpt1a* and *Acox1* in OA-exposed primary hepatocytes (Figure 3B). Given that the inhibition of lipid droplet formation, TG levels, and the lipogenesis genes mRNA expression conferred by MBC was similar in the primary murine hepatocytes and SK-HEP-1 cells, therefore we used the SK-HEP-1 cells in further mechanistic assessments.

We determined the protein expression levels of FASN, SREBP-1c, ACC1, and PPAR-α by Western blot in 0.5 mM of OA and MBC treated SK-HEP-1 cells. Coordinated with that of mRNA, the protein levels of FASN, SREBP-1c and ACC1 were reduced, and the expression of PPAR-α was increased by MBC in a dose-dependent (20, 40, 60 and 80 µM) manner (Figure 3C). Consistently, the results between the protein level and the gene level were almost the same (Figure 3A,C).

### 2.3. Effect of MBC on AMPK Activation in OA-Treated SK-HEP-1 Cells

We determined the AMPK phosphorylation in SK-HEP-1 cells after 0.5 mM of OA treatment for 48 h. The results revealed that the phosphorylation of AMPK (Thr172) was reduced by ~32% (Figure 3D). The ratio of phosphorylated p-AMPK to AMPK was decreased in SK-HEP-1 cells (Figure 3D). However, the phosphorylation of AMPK protein was increased by MBC in a concentration-dependent (20, 40, 60 and 80 µM) manner after a treatment period of 48 h (Figure 3D). Moreover, treatment with MBC dose-dependently increased the p-AMPK:AMPK ratio as compared to those in the control group (Figure 3D).

### 2.4. Effect of MBC on Inflammation in OA-Treated SK-HEP-1 Cells

**Figure 4:**
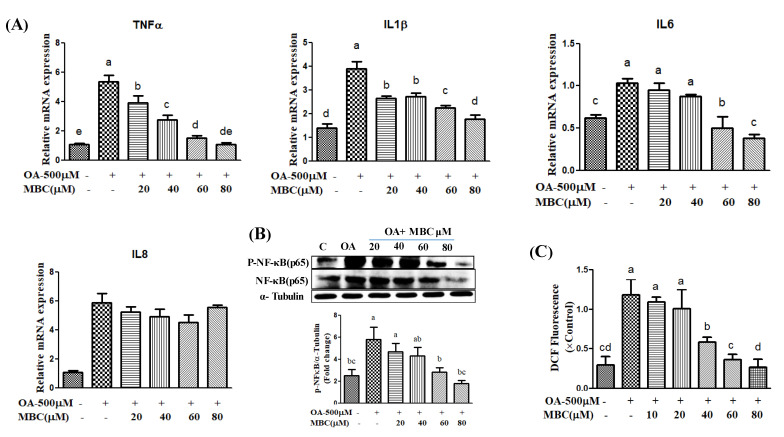
Effect of methyl brevifolincarboxylate (MBC) on inflammatory mediator mRNA and ROS production in OA-treated SK-HEP-1 cells. (**A**) Effect of MBC on secretion of inflammatory mediators determined by RT-PCR. (**B**) Effect of MBC on protein expression of NF-κB (p65) determined by Western blot. (**C**) Intracellular ROS level was analyzed with 2′,7′-dichlorodihydrofluorescein (DCF) using a fluorescence reader at an excitation wavelength of 485 nm and an emission wavelength of 535 nm. Cells were incubated with 0.5 mM of OA in the presence or absence of different concentrations of MBC for 48 h. All results were presented as mean ± SEM of three independent experiments. Data bars with similar letters were not significantly different (*p* ≤ 0.05).

Inflammation plays an important role in the pathogeneses of NAFLD. We evaluated the effects of MBC on the levels of pro-inflammatory factors in SK-HEP-1 cells after 0.5 mM of OA-treatment for 48 h. As expected, our RT-PCR results showed that OA-treatment increased the levels of inflammation-related gene expression of *TNF-α*, *IL-6*, *IL-8* and *IL-1β* in SK-HEP-1 cells (Figure 4A). The MBC dose-dependently (20, 40, 60 and 80 µM) attenuated the expression of *TNF-α*, *IL-6*, *IL-8*, and *IL-1β* (Figure 4A). To investigate the possible anti-inflammatory mechanism of MBC, we determined the expression of NF-κB in SK-HEP-1 cells after 0.5mM of OA-treatment for 48 h. Our Western blot results showed that the expression of NF-κB was noticeably elevated in OA-treated cells. The MBC treatment for 48 h, dose-dependently (20, 40, 60 and 80 µM) decreased the expression of NF-κB (Figure 4B). We used the peroxide/redox sensitive fluorescent probe DCFH-DA to measure total intracellular ROS levels. Our results showed that the levels of ROS were increased in 0.5 mM of OA-treated SK-HEP-1 cells (Figure 4C) whereas the MBC treatment dose-dependently (40, 60 and 80 µM) decreased the levels of ROS (Figure 4C).

## 3. Discussion

It is well acknowledged that natural phytochemicals from herbal preparations and dietary products such as flaxseed, cinnamon, silybin, soy, curcumin, licorice root, epigallocatechin-3-gallate, theaflavin and fish-derived proteins or hydrolysates have potential anti-NAFLD properties [14,15]. *Phyllanthus urinaria* is an important traditional herbal medicine widely used to treat various diseases including liver protection, diabetes, hepatitis and jaundice [16]. For example, *P. urinaria* capsules attenuated steatohepatitis through reduced oxidative stress, inflammation and lipid accumulation [17,18]. In this study, our results demonstrated that the *P. urinaria* major active metabolite MBC attenuated the excessive lipid accumulation and TG levels through amelioration of lipid metabolism signaling components.

The human liver is an important body organ that is responsible for maintenance of lipid metabolism through both de novo lipogenesis and lipid oxidation [19]. Hepatocytes are the main cells in the liver, and are responsible for the major physiological and metabolic functions of the liver. Hepatocytes take up and use large amounts of lipids such as free fatty acids. However, excessive synthesis of lipids exceeding their secretion leads to hepatic steatosis or lipid accumulation in the liver (i.e., primary stage NAFLD) [3]. Previous studies indicate that OA can affect hepatic lipid metabolism and OA-induced cells that are widely used for NAFLD-type induction in vitro [3]. Excessive intracellular TG level is one of the indicators of NAFLD [3]. In the present study, SK-HEP-1 cells and primary murine hepatocytes were incubated with OA to induce cellular steatosis. These two cell lines were chosen because they have been frequently used in the literature to establish the in vitro model of NAFLD [1]. Our results showed that the non-toxic concentrations of MBC dampened the TG levels in OA-treated SK-HEP-1 cells and primary murine hepatocytes (Figure 1D,E). Lipid accumulation in specialized subcellular organelles called lipid droplets (LDs) is another indicator of NAFLD [3]. The LDs formation starts by transformation of fatty acids and free cholesterol into neutral lipids [3]. In this study, Oil Red O and H&E staining results showed that MBC treatment attenuated the OA-induced LD formation in both the SK-HEP-1 cells and primary murine hepatocytes (Figure 2).

Lipid accumulation in HCC cells is caused by elevated de novo lipogenesis. Among the lipogenesis enzymes, FASN plays an important role in NAFLD pathogenesis through increased fatty acid production [6]. On the other hand, the two ACC isoforms ACC1 and ACC2 are distinct, and are encoded by different genes [20]. The ACC1 is highly expressed in lipogenic tissues such as liver and adipose tissue, and ACC2 shows elevation of its expression in heart, muscle and liver cells [20]. ACC1 catalyzes the carboxylation of acetyl-CoA into malonyl-CoA, that acts as a substrate for synthesis of saturated fatty acid, C16 or palmitic acid (PA)] [20]. The enzymes FASN and ACC1 catalyze the synthesis of PA that can undergo structural modifications to generate other fatty acid that causes NALFD pathogenesis [20]. Therefore, the inhibitors of FASN and ACC1 have therapeutic significance in the treatment of NAFLD [21]. For example, a recent clinical trial of NAFLD/NASH reports that the ACC1 inhibitor, GS-0976 decreases liver steatosis [22]. The FASN inhibitor, FT-4101 reduces hepatic de novo lipogenesis and has entered into clinical trials of NAFLD [23]. Additionally, it is acknowledged that various natural FASN and ACC1 inhibitors including luteolin, curcumin and resveratrol attenuate NAFLD in various in vitro and in vivo models [8,14]. In this study, our results demonstrated that MBC reduced both the mRNA and protein expression of FASN and ACC1 in vitro (Figure 3). To our knowledge, we report here for the first time that MBC was an inhibitor of FASN and ACC1 expression.

SREBP-1c is a transcription factor and an elevation of its expression levels leads to excessive TG accumulation that contributes to the development of NAFLD pathogenesis [6,20]. The SREBP-1c positively regulates the gene expression of the lipogenic enzymes, FASN and ACC1 [6]. In this study, MBC reduced the expression of SREPB-1c in vitro in addition to its inhibition of potential against FASN and ACC1 (Figure 3). Thus, our results indicated that the reduced lipid accumulation and TG levels in OA and MBC-treated SK-HEP-1 cells and primary murine hepatocytes was associated with decreased SREBP-1c and its downstream targets, FASN and ACC1.

PPARs are a group of ligand-activated transcription controlling molecules that modulate lipid metabolism through the regulation of not only lipogenesis genes but also genes involved in inflammation [24]. PPAR-α target genes ACOX1 and CPT1a are involved in β-oxidation in the liver [24]. Therefore, PPAR-α and its target genes agonists may useful for amelioration of NAFLD through their increased lipid oxidation properties as demonstrated with the well-known PPAR-α activator, saroglitazar [25]. In our study, MBC promoted the activity and expression of PPARα. Our results support previous reports that demonstrate inhibition of NAFLD pathogenesis including the down-regulation of lipogenesis markers, SREBP-1c, FASN and ACC1, and up-regulation of the lipid oxidation marker, PPARα [24]. The PPARα regulates the expression of CPT1a and ACOX1 levels and elevates PPARα expression concomitantly with the increased expression of CPT1 and ACOX1 [24]. In this study, our results showed that MBC increased the mRNA levels of *Cpt1a* and *Acox1* in OA-treated SK-HEP-1 cells and primary murine hepatocytes (Figure 3A,B).

The AMPK plays a key role as a master regulator of lipid metabolism, and its pharmacological activation has therapeutic potential against NAFLD [7]. The optional NAFLD commercial drug, metformin has therapeutic potential by targeting the AMPK signaling pathway [7]. Activation of AMPK through Thr172 phosphorylation reduces the expression of the lipogenesis factor, SREBP-1c and its downstream targets, FASN and ACC1 [7]. A number of studies identified that various natural products improve hepatic steatosis in NAFLD through activation of the AMPK pathway. For example, silibinin inhibits de novo lipogenesis and promote lipid oxidation of NAFLD through activation of the AMPK pathway [26]. A steroidal saponin dioscin, isolated from various kinds of vegetables and herbs, attenuates lipid metabolism in NAFLD through the AMPK pathway [27]. Licochalcone A reduces lipogenesis and increases β-oxidation in hepatocytes through the AMPK pathway [28]. Our results showed that MBC treatment increased AMPK phosphorylation and reduced de novo lipogenesis (reduced mRNA and protein for FASN, SREBP-1c and ACC1) and increased lipid oxidation (increased mRNA and protein for PPAR-α and ROS concentration) molecules.

**Figure 5:**
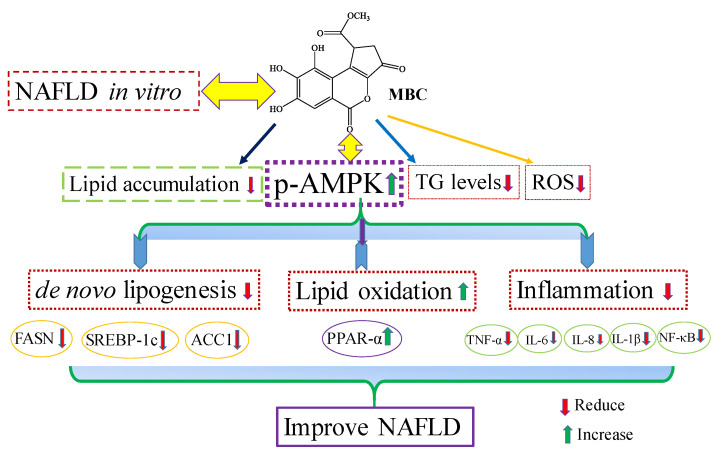
Schematic presentation of methyl brevifolincarboxylate (MBC) effect on NAFLD in vitro.

Lipid accumulation in hepatocytes leads to increased oxidative stress and mitochondrial dysfunction that induces the inflammatory response through the elevated secretion of pro-inflammatory cytokines [19]. Various pro-inflammatory interleukin type cytokines such as IL-6, IL-8, and IL-1β and TNF-α levels were elevated in the progression of NAFLD [19]. The TNF-α is secreted by liver hepatocytes and Kupffer cells; TNF-α is a main factor in the progression of NAFLD [19]. During NAFLD progression, the inflammatory mediators IL-6, IL-8, IL-1β and TNF-α are regulated by a critical nuclear transcription factor, NF-κB [19]. Previous studies have reported that activation of NF-κB leads to an increased level of inflammation [19]. Therefore, suppression of inflammation through production of reduced inflammatory mediator’s has therapeutic benefit in the treatment of NAFLD [19]. In the present study, MBC treatment reduced *TNF-α*, *IL-6*, *IL-8* and *IL-1β* levels, in addition to reduced expression of NF-κB (Figure 4). Previous studies indicate that the natural compounds, baicalin [29], naringenin [30] and chicoric acid isolated from *Crepidiastrum denticulatum* [31] reduced NAFLD through attenuation of hepatic inflammation. Oxidative stress is the net result of an imbalance between the ROS production and antioxidant defense, which leads the liver damage in the progression of NAFLD [1,2,3]. Activation of the NF-κB signaling pathway induces ROS production through regulation of lipid metabolism molecules [1,2,3]. Our results showed that MBC reduced the ROS production in OA-treated SK-HEP-1 cells. Accordingly, the present study demonstrated that MBC protects against NAFLD at least in part through increased AMPK phosphorylation and there by reduced SREBP-1c-mediated de novo lipogenesis and increased PPAR-α-dependent lipid oxidation. Furthermore, MBC also attenuated secretion of the inflammatory cytokines through reduced NF-κB expression. The schematic presentation of the effect of MBC on lipid metabolism molecules and inflammation was presented as in Figure 5. We confirmed for the first time that the polyphenolic compound MBC attenuated NAFLD in vitro by decreased hepatic lipid synthesis and reduced inflammation through the AMPK and NF-κB signaling pathways.

The limitations of this study were that it did not verify the anti-inflammatory effect of MBC in Kupffer cells and macrophages, which are known the cells involved in inflammatory response of the liver. Additional, in vivo studies were required to elucidate the anti-NAFLD activity of MBC to continue its development as a therapeutic agent against NAFLD. The bioavailability and metabolism of phytochemicals is fundamental to understand their impacts on human health. The metabolism of phytochemicals depends on the individual’s digestive ability, membrane transporters, metabolizing enzymes and gut microbiota. The exogenous compounds (phytochemicals) are metabolize in gut microbiota, and phase I and phase II metabolism through oxidation, hydration, demethylation, hydrogenation, and SH_2_ addition, as well as different glucuronide/sulfate conjugates [32]. A recent study reported that the structurally similar compounds to MBC are orally bioavailable and undergo gut microbiotic metabolism (dehydroxylation and reduction), and phase II metabolism (glucuronidation and sulfation) in healthy humans [33]. Further in vivo studies are need to investigate the bioavailability, distribution and metabolism of MBC. However, the results of our study are useful for the identification of novel therapeutic agents against NAFLD.

## 4. Conclusions

This study demonstrated that the effect of *Phyllanthus urinaria* compound methyl brevifolincarboxylate (MBC) on fatty acid-induced NAFLD in vitro. The results suggest that MBC attenuated the dysregulated lipid accumulation and TG levels. The underlying molecular mechanism was associated with reduced activity and expression of de novo lipogenesis molecules and increased lipid oxidation factors. The MBC mechanism also decreased inflammatory molecules and ROS production through suppression of the AMPK and NF-κB signaling pathways.

## 5. Materials and Methods

### 5.1. Reagents and Chemicals

Oleic acid and HyClone Dulbecco’s modified Eagle’s medium (DMEM)-high glucose for cell culture were obtained from GE Healthcare Life Sciences (Logan, UT, USA). Primary antibodies against SREBP-1c (AF6283, RRID:AB_2835134), ACC1 (AF7864, RRID:AB_2844228), FASN (DF6106, RRID:AB_2811172), and PPAR-α (AF5301, RRID:AB_2837786), CPT1a (DF12004, RRID:AB_2844809), ACOX1 (DF12046, RRID:AB_2844851) were obtained from Affinity Biosciences (OH, USA). Antibodies against p-AMPK (T172) (#2535), AMPK (#2532), and NF-κB (#3033) were obtained from Cell Signaling Technology, Inc., (Boston, MA, USA). β-Actin mAb (#4970) was obtained from Cell Signaling Technology, Danvers, MA, USA. Penicillin, streptomycin, fetal bovine serum (FBS), and amphotericin B (PSA) were obtained from Biological Industries (Beit Haemek, Israel). Fatty-acid low BSA was obtained from Scientific Biotech Corp. (Taipei, Taiwan). Superscript II reverse transcriptase was purchased from Thermo Scientific RevertAid RT Kit (Thermo Fisher Scientific, Waltham, MA, USA). All reagents used were of analytical grade.

### 5.2. Extraction and Isolation of MBC

The pure compound MBC was isolated from the whole plant of *P. urinaria* following the extraction and isolation procedures as reported previously [9]. Briefly, the air dried whole plants of *P. urinaria* was pulverized into powder and extracted three times with 95% EtOH at room temperature for 24 h. The ethanol extracts were combined and concentrated under reduced pressure at 50 °C to yield a yellowish residue. The residue was purified using silica gel column chromatography. The mobile phase consisting of a gradient of n-hexane/ethyl acetate ranging from 9:1 to 3:7. The fractions obtained from column chromatography were monitored using thin layer chromatography (TLC). Similar fractions were combined to give five fractions (F1–F5). Further purification F2 resulted in the pure compound methyl brevifolincarboxylate (MBC, Figure 1A). The purity of the compound (>95%) was confirmed by its NMR spectral data [9].

### 5.3. Animals

Male C57BL/6 mice (8-week-old, Specific pathogen-free grade, weight 18–22 g), were provided by the National Laboratory Animal Center (NLAC) (Nangang, Taipei, Taiwan). Mice maintained on a 12-h/12-h light/dark cycle in a pathogen-free animal facility at the Department of Animal Science and Technology, National Taiwan University. After 1 week of acclimatization, the mice were used for the experiment. The experiment was performed by following guidelines of the Animal Care Ethics Committee of National Taiwan University.

Primary murine hepatocytes were isolated from 8-week-old C57BL/6 mice, using a collagenase perfusion method as described previously [34]. After liver dissociation, the cells were filtered through a 45-μm cell strainer, and washed three times at 50× *g* for 2 min. The cells were suspended in 40% Percoll Plus solution (GE Healthcare, Tokyo, Japan), and centrifuged at 50× *g* for 20 min at 4 °C to further purify the hepatocytes and enrich the viable cells. In this study, 40% Percoll solution was prepared using 4 mL of 100% Percoll (Stock Concentration) and 6 mL of 10XPBS (Final: 10 mL of 40% Percoll solution).

### 5.4. Cell Culture and Treatment

The human hepatocarcinoma cell line, SK-HEP-1 was cultured in high glucose-Dulbecco’s Modified Eagle Medium (HG-DMEM; HyClone, GE Healthcare Life Sciences, Logan, UT, USA). All media were supplemented with 10% fetal bovine serum (Thermo Fisher Scientific, Waltham, MA, USA) and 1% 100 units/mL penicillin, 100 mg/mL streptomycin and 0.025 mg/mL amphotericin B. Primary hepatocytes were cultured in William’s Medium E (supplemented with 5% fetal bovine serum, 100 units/mL penicillin, 100 μg/mL streptomycin, 1 × ITS and 5 nM dexamethasone). Cells were incubated in 12-well plates at an initial density of 1 × 10^5^/well (SK-HEP-1) and 2.5 × 10^5^/well (primary hepatocytes), in a 5% CO_2_ humidified atmosphere incubator at 37 °C. When the cells reached 90% confluence, they were starved with serum-free DMEM for 24 h. For fatty hepatocyte model, supplemented with OA was used to induce lipid accumulation. Standard compounds OA and MBC were dissolved in ethanol, diluted to the desired concentrations with serum free DMEM medium prior to treatment of cells. The final concentration of ethanol was always <0.01%, a concentration that does not affect the experimental results.

### 5.5. Cytotoxicity Assay

Cell cytotoxicity was determined using an Alamar Blue assay. SK-HEP-1 cells and primary murine hepatocytes were seeded in 96-well plates (SK-HEP-1: 1 × 10^5^ cells/well; primary hepatocytes: 2.5 × 10^5^ cells/well). Different concentrations of MBC (1–500 μM) were added and cells were incubated cells at 5% CO_2_ in air at 37 °C. After 48 h, the cells were treated with 10% Alamar Blue reagent followed by a 4h incubation at 37 °C. The fluorescence absorbance was read using an ELISA reader at 570/600 nm.

### 5.6. Oil Red O Staining, and Hematoxylin and Eosin (H&E) Staining Assay

The intracellular lipid accumulation was determined using Oil Red O staining, and H&E staining assay. SK-HEP-1 cells and primary murine hepatocytes were seeded into 12-well culture plates and incubated with 0.5 mM of OA and in the presence or absence of different concentrations of MBC for 48 h. After 48 h medium was discarded and plates were gently washed with PBS. Cells were fixed with 10% formaldehyde for 1 h, the fixative was removed and cells were washed with water followed by 60% isopropanol. The cells were covered with Oil Red O working solution and rotated for 1 h. The stain was discarded and the cells were washed with water until no stain was removed. The hematoxylin solution was added to the cells for 1 min, and then cells were washed with water. The lipid droplets were photographed under a microscope.

### 5.7. ROS Assay

The levels of intracellular reactive oxygen species (ROS) were measured using the DCFDA (2′,7′-dichlorodihydrofuorescein diacetate) assay. The SK-HEP-1 cells were plated into 96-well plates and incubated overnight at 37 °C. When the cells reached 90% confluence, they were starved for 24h with serum-free DMEM, and then cells were treated with OA (0.5 mM) and different concentrations of MBC (10 to 80 μM) and incubated for 48 h. Cells were stained with 10 μM DCFDA medium for 30 min at 37 °C. After 30 min incubation fluorescence was measured using a fluorescence reader at an excitation/emission wavelengths of 485/535 nm.

### 5.8. Triglyceride Quantification

Intracellular triglycerides were determined using a triglyceride assay kit (Randox Laboratories Ltd., Crumlin, UK). The SK-HEP-1 cells and primary murine hepatocytes were cultured in 12-well plates, treated with 0.5 mM of OA and different concentrations of MBC. After 48 h incubation, the culture medium was removed, and cells were twice washed with cold PBS. The cells were homogenized with 5% NP–40/ddH_2_O using TissueLyser II (Qiagen, Venlo, The Netherlands), then using a sterile scraper, gently scrap the cell colonies and transferred. The homogenates were transferred into 1.5 mL Eppendorf tubes, slowly heated to 80–100 °C, and centrifuged for 2 min at top speed (4 °C; 3000× *g*) using a microcentrifuge, and then supernatants were collected. The intracellular concentration of TG was determined on the supernates according to the manufacturer instructions. The TG was normalized to protein concentration.

### 5.9. RNA Extraction and Quantitative Real-Time PCR Analysis

Then genomic DNA was removed from the samples using TURBO DNA-free kit (Invitrogen, Carlsbad, Carlsbad city, CA, USA), and reverse transcribed to cDNA using a High Capacity cDNA Reverse Transcription kit (Thermo Fisher Scientific, Waltham, MA, USA). Total RNA was extracted from SK-HEP-1 cells and primary murine hepatocytes using the GENEzol^TM^ Reagent (Geneaid Biotech, Ltd., New Taipei City, Taiwan). The concentration and purity of RNA was measured with NanoDropTMOne spectrophotometer (Thermo Fisher Scientific, Waltham, MA, USA) at 260/280 nm. Quantitative real-time PCR reactions were performed via StepOnePlus™ Real-Time PCR System (Applied Biosystems, Foster City, CA, USA) with a DyNAmo Flash SYBR Green High-ROX detection Kit (Finnzymes, Espoo, Finland). The RT-PCR conditions were: for polymerase activation at 95 °C for 2 min and the 40 cycles denaturation at 95 °C for 10 s followed by 30 s of annealing/extension at 60 °C. The specific primer sequences used were listed in Table 1. Gene expression was calculated using Threshold cycle (Ct) values. The internal control β-actin mRNA was measured in each sample and target gene mRNA levels were expressed as a ratio with reference to the expression of β-actin.

### 5.10. Protein Extraction and Western Blot Analysis

Cells were washed with cold PBS before harvesting cell lysates. Cell lysates were prepared in RIPA lysis buffer containing a protease and phosphatase inhibitors (Thermo Fisher Scientific, Waltham, MA, USA). The samples were homogenized using a mortar and pestle on dry ice, and were spun at 14,000× *g* for 30 min. The supernatant fractions were assayed for protein using the BCA protein assay (Thermo Fisher Scientific, Waltham, MA, USA). Aliquots of cell lysates containing equal quantities of protein (20 µg/lane) were separated by using 10% SDS-PAGE. Separated proteins were transferred onto methanol-activated PVDF membranes (PerkinElmer, Inc., Waltham, MA, USA). The buffer for incubation with antibodies was composed of 25 mM of Tris (pH 7.4), 150 mM of NaCl, 0.1% Tween of 20, and 5% skim milk with gentle shaking for 1 h. The membranes were incubated with primary antibodies against SREBP-1c, ACC1, FASN, PPAR-α, AMPK, pAMPK, and NF-κB or mouse monoclonal antibody against α-tubulin over night at 4 °C. The membranes were then washed using TBST (tris-buffered saline with 0.1% Tween^®^ 20 detergent) buffer three times for 10 min each, and then were incubated with horseradish peroxidase-conjugated secondary antibodies (Thermo Fisher Scientific, Inc.) at 4 °C for 1 h. Signals were detected using the ChemiDoc Touch Imaging System (Bio-Rad Laboratories, Inc., Santa Clara, CA, USA). The quantitation of Western blot bands was conducted by comparison against α-tubulin using Image Lab software (Bio-Rad Laboratories, Inc., Santa Clara, CA, USA).

### 5.11. Statistical Analysis

Statistical data were analyzed using GraphPad Prism (GraphPad Software, Inc., San Diego, CA, USA) and performed one way analysis of variance (ANOVA) followed by Tukey’s multiple comparison. The data were represented as mean ± SEM or mean ± SD as indicated. *p* ≤ 0.05 was considered statistically significant.

## Figures and Tables

**Table 1:** Sequences for real-time PCR primers.

Name	Forward Primer (5′→3′)	Reverse Primer (5′→3′)
Human		
*Srebp1c*	CTGTTGGTGCTCGTCTCCTTGG	AGCAGGTGACGGATGAGGTTC
*Acc1*	ACATGCTTCAAAGGTCCAGC	TCCCCCAAAGCGAGTAACAA
*Fasn*	ACAGGGACAACCTGGAGTTG	CTGTGGTCCCACTTGATGAGT
*Pparα*	GCAGAAACCCAGAACTCAGC	ATGGCCCAGTGTAAGAAACG
*Cpt1a*	GCAGCGTTCTTTGTGACGTT′	AGGAGTGTTCAGCGTTGAGG
*Acox1*	CCCATAAGCCTTTGCCAGGA	GGCTTCACCTGGGCATACTT
*TNF-α*	AGCCTCTTCTCCTTCCTGAT	AAGATGATCTGACTGCCTGG
*IL-1β*	AACAGGCTGCTCTGGGATTC	AGATTCGTAGCTGGATGCCG
*IL-6*	AATGAGGAGACTTGCCTGGTG	CTGGCGATTTGTGGTTGGGTC
*IL-8*	CCAGGAAGAAACCACCGGA	GAAATCAGGAAGGCTGCCAAG
*β-actin*	GAAGATCAAGATCATTGCTCCTC	CTAAGTCATAGTCCGCCTAGAAG
Mouse		
*Fasn*	GGAGGTGGTGATAGCCGGTAT	TGGGTAATCCATAGAGCCCAG
*Acc1*	TAATGGGCTGCTTCTGTGACTC	CTCAATATCGCCATCAGTCTTG
*Srebp1c*	GGAGCCATGGATTGCACATT	GCCCGGGAAGTCACTGT
*Pparα*	TGCCTTAGAACTGGATGAC	ATCTGGATGGTTGCTCTG
*Cpt1a*	AACAGCAAGATAGGCATAA	TGTCCATCCTCTGAGTAG
*Acox1*	CAGGAAGAGCAAGGAAGT	AGAGAATATAAGAGACACAGGTT
*β-actin*	TGTTACCAACTGGGACGACA	CTTTTCACGGTTGGCCTTAG
